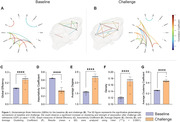# Activation of the glutamate transport in astrocytes via GLT‐1 reshapes the glutamatergic brain network

**DOI:** 10.1002/alz.092645

**Published:** 2025-01-09

**Authors:** Gabriel Colissi Martins, Christian Limberger, Guilherme G. Schu Peixoto, Antonio Aliaga, Arturo Aliaga Aliaga, Pedro Rosa‐Neto, Eduardo R. Zimmer

**Affiliations:** ^1^ Universidade Federal do Rio Grande do Sul, Porto Alegre, Rio Grande do Sul Brazil; ^2^ McGill University, Montreal, QC Canada; ^3^ Brain Institute of Rio Grande do Sul ‐ Pontifícia Universidade Católica do Rio Grande do Sul, Porto Alegre, Rio Grande do Sul Brazil

## Abstract

**Background:**

Glutamate is the main excitatory neurotransmitter in the brain, acting through ionotropic and metabotropic receptors, such as the neuronal metabotropic glutamate receptor 5 (mGluR5). The radiotracer [^11^C]ABP688 binds allosterically to the mGluR5, providing a valuable tool to understand glutamatergic function. We have previously shown that neuronal [^11^C]ABP688 binding is influenced by astrocyte activation. Specifically, the activation of glutamate transport in astrocytes via glutamate‐transporter‐1 (GLT‐1) results in an elevated [^11^C]ABP688 binding within the ventral anterior thalamus, but no changes in other regions. However, whether changes at the network level occur remains elusive. We evaluated Glutamatergic Brain Networks (GBNs) before and after activating astrocytes.

**Method:**

Micro‐PET acquisitions were performed in adult Sprague‐Dawley rats (n = 5) at baseline and after a pharmacological challenge to activate astrocytes. Before the scan, rats received either saline (vehicle) or ceftriaxone (CEF, 200mg/kg), an antibiotic that induces GLT‐1 activation. Then, [^11^C]ABP688 was administered i.v., followed by a 60‐minute dynamic scan. The nondisplaceable binding potential (BPND) was estimated using the simplified reference tissue model, with the cerebellum as a reference region. [^11^C]ABP688 BPND was extracted for 12 volumes of interest. GBNs based on [^11^C]ABP688 regional BPND were assembled with a multiple sampling scheme in MATLAB (p‐value < 0.05).

**Result:**

Astrocyte activation induced GBN hyperconnectivity after astrocyte activation, with the strength of association between the cerebral hemispheres, such as in the frontal and parietal cortices (Figure 1, A‐B). After GLT‐1 activation, the connections were diffused, including regions that were not part of the network at baseline, such as the cerebellum. Graph measures show a significant increase in global efficiency, average degree, density, and average clustering coefficient (Figure 1, C‐G). These measures indicate an increase in connections and network cohesiveness. The reduction in assortativity can be related to the new regions on the GBN after the challenge.

**Conclusion:**

Our results show that GBNs assessed with [^11^C]ABP688 are sensitive to astrocyte activation. Alterations in glutamate levels, as observed in dysfunctions like glutamatergic excitotoxicity present in AD, could disrupt the cohesion of the glutamatergic network, leading to brain dysfunction.